# Exogenous ABA combined with cytokinin enhances drought resistance in *Ziziphus jujuba* Mill. var. *spinosa* by regulating xylem development and zeatin signaling pathway

**DOI:** 10.3389/fpls.2026.1901882

**Published:** 2026-07-30

**Authors:** Shuhui Zhou, Meng Li, Mingyu Jiang, Wei Zheng, Jin Yang, Chenghui Li, Ruixue Wang, Yao Zhao

**Affiliations:** 1 College of Resources and Environment, Linyi University, Linyi, China; 2 Shandong Provincial Forestry Protection and Development Service Center, Jinan, China

**Keywords:** drought stress, ABA, cytokinin, *Ziziphus jujuba* Mill. var. *spinosa*, zeatin signaling

## Abstract

Drought is a decisive abiotic constraint limiting the growth and yield of jujube, its alleviation is orchestrated by a complex hormonal network that governs the tree’s drought-resistance responses. Based on preliminary evidence that abscisic acid (ABA) and cytokinin play coordinated roles in drought adaptation, this study aimed to clarify their individual and combined effects on regulating drought resistance in sour jujube. Phenotypic responses, anatomical alterations, endogenous hormone profiles, and the expression levels of genes involved in the zeatin biosynthesis pathway after exogenous hormone spraying under drought stress were comprehensively analyzed. The results demonstrated that ABA and cytokinin significantly modulated drought adaptation of sour jujube across multiple aspects. Compared with the control group, ABA spraying xylem vessels number in leaf veins and roots, shortened stem internodes, and elevated the content of salicylic acid (SA), thereby enhancing drought resistance. In contrast, spraying cytokinin treatment increased drought sensitivity, expanded root xylem vessel diameter, and induced a slender stem and increased auxin content. Combined application of ABA and cytokinin was found to further increase vessel number and diameter in leaves and roots, improve branching density, and significantly increase SA, ABA, and jasmonic acid (JA) levels compared with single application of ABA and cytokinin. These factors may collectively enhance the drought resistance of sour jujube. Through weighted gene co-expression network analysis (WGCNA), candidate genes such as *CGLD27*, *GNAT10*, and *LOC107415885* were obtained. Further analysis found that, both ABA and cytokinin inhibited zeatin biosynthesis, while accelerated trans-zeatin metabolism and delayed cis-zeatin turnover. Spraying of cytokinin enhanced isopentenyladenine degradation, which may reduce resistance, whereas downregulation of *ZjZOG1*, *ZjZOG2*, and *ZjZOG3* may promote resilience. These findings provide a preliminary mechanistic framework for the role of ABA and cytokinin in drought adaptation of sour jujube.

## 1 Introduction

Climate change is significantly increasing the frequency and intensity of drought stress, leading to reduced crop yields and quality, thereby posing a severe threat to global agricultural production and food security (Krishnamurthy et al., 2022). Plants subjected to drought stress undergo structural and functional changes that compromise their survival and growth (Aberkane et al., 2021). To cope with water scarcity, plants have evolved adaptive strategies, collectively termed drought resistance, which include preventing water loss, optimizing water allocation to vital organs, maintaining cellular hydration, and ensuring survival under prolonged drought (Gupta et al., 2020). Previous studies have shown that drought-resistant plants exhibit superior water-use efficiency by regulating the number, diameter, and distribution of xylem vessels compared to drought-sensitive species (Mao et al., 2025). Additionally, these plants enhance photosynthetic efficiency, antioxidant capacity, and osmolyte accumulation to sustain growth under water deficit (Du et al., 2020).

Phytohormones play pivotal roles in plant growth, development, and stress responses, serving as key inducers of drought resistance (Luo et al., 2021). Under abiotic stress, the exogenous application of phytohormones—such as abscisic acid (ABA), salicylic acid (SA), and auxin—at appropriate concentrations can effectively mitigate stress-induced damage. Among these, ABA is widely recognized as the most critical hormone in drought adaptation (Baek et al., 2023). Upon drought onset, ABA rapidly accumulates and orchestrates stress responses by regulating stomatal closure, root growth, and the biosynthesis of protective metabolites (Chen et al., 2022). However, numerous studies have demonstrated that while ABA enhances drought adaptability, it concurrently induces plant dwarfism (Liu et al., 2022; Wang et al., 2024). Cytokinin, initially identified as potent inducers of cell division in tissue culture, are now established as key regulators of the cell cycle (Kakimoto, 2003). Foliar application of 6-benzylaminopurine (6-BA) and kinetin (KT) typically induces narrow leaves and elongated stems (Kieber and Schaller, 2018). In the context of tissue culture in woody plants, the combined application of 6-BA and KT has been shown to significantly enhance bud induction rates, thereby promoting growth (Wang et al., 2023). Notably, crosstalk between distinct phytohormones, such as ABA and auxin (Yao et al., 2025) or ABA and SA (Singh et al., 2024) enables coordinated drought responses. Zhou et al. (2016) reported that ABA and cytokinin can jointly regulate modulate photosynthetic efficiency, and their combined treatment can enhance photosynthetic activity in *Zea mays*, thereby improving drought tolerance. During drought stress, declining cytokinin levels coupled with ABA accumulation often suppress seedling growth, impair photosynthesis, and accelerate leaf senescence. Conversely, cytokinin supplementation antagonizes these effects, promoting growth and cotyledon greening (Foyer et al., 2016). Thus, while ABA is indispensable for drought resistance, cytokinin appears to counteract the effects of growth inhibition and senescence. However, it remains unclear whether the co-application of ABA with cytokinin could mitigate the dwarfing effects observed under drought stress and further coordinate plant water stress adaptability and growth performance. This potential interaction warrants further investigation to elucidate the underlying mechanisms and optimize strategies for improving stress resilience in woody species.

*Ziziphus jujuba* Mill. var. *spinosa*, a species of the Rhamnaceae family and the *Ziziphus* genus, is a wild fruit tree native to China. It exhibits high economic value as various plant parts—including fruits, leaves, pollen, seeds, roots, and thorns—are utilized in traditional medicine (Wu et al., 2022). Due to exceptional drought resistance and adaptability to nutrient-poor soils, sour jujube is widely employed as a rootstock for more than 900 jujube varieties (Hua et al., 2022; Liu et al., 2020). Understanding the molecular mechanisms underlying its drought adaptation is therefore crucial for breeding stress-resistant rootstock varieties. Our previous studies have suggested that ABA and cytokinin may coordinately regulate xylem development in autotetraploid sour jujube under drought conditions (Li et al., 2021). However, the specific pathways through which these hormones modulate drought resistance remain elusive. Therefore, we comprehensively assessed the effects of exogenous ABA and cytokinin (6-BA and KT) treatment on sour jujube seedlings by analyzing their phenotypic, anatomical, physiological, and molecular responses to drought stress. Our findings could not only provide critical insights into the hormonal regulation of xylem plasticity in sour jujube during water deficit but also identify key signaling pathways involved in drought adaptation.

## 2 Materials and methods

### 2.1 Plant materials and treatments

Seedlings of sour jujube were cultivated in the plant growth chamber under controlled conditions (26°C, 16/8 h light/dark photoperiod). Seven-week-old seedlings with uniform growth status were selected for drought stress. For the drought stress treatment, watering was withheld until the soil water content reached 40% of field capacity, as determined by the weighing method.

The hormone treatments were identical to those reported in Li et al. (2026), which focused on the phenylpropanoid pathway. The present study analyzed the same samples to investigate xylem structural modifications and hormone responses under the same treatments. In short, 5 mg·L^-1^ ABA was identified as the optimal concentration for drought-stress mitigation. For the cytokinin treatment, 50 mg·L^-1^ 6-benzylaminopurine (6-BA) and 100 mg·L^-1^ kinetin (KT) was selected. During the drought period, four treatment groups were established: (1) DCK (drought control): seedlings sprayed with distilled water; (2) DA: seedlings treated with ABA solution (5 mg/L); (3) DC: seedlings treated with a combination of 6-BA (100 mg/L) and KT (50 mg/L); (4) DAC: seedlings treated with a mixture of ABA (5 mg/L), 6-BA (100 mg/L) and KT (50 mg/L). All solutions contained 0.2% (v/v) Tween-20 as a surfactant to ensure optimal leaf adhesion. Treatments were applied by foliar spraying every 12 h for 14 consecutive days, with three biological replicates per treatment and each replicate containing 10 plants. Subsequently, the height of the plants and the aboveground biomass were measured respectively. For anatomical structure observation, leaves, stems, main roots, and lateral roots were collected from three representative seedlings per biological replicate. Mature leaves were pooled and used for physiological analysis, endogenous hormone analysis and gene expression analysis.

### 2.2 Analysis of physiological characteristics

Fresh leaves were immersed in absolute ethanol and kept in the dark for 24 h to extract chlorophyll (Wang et al., 2006). The absorbance of the extract was measured at 665 nm and 649 nm to calculate the chlorophyll content. Relative water content (RWC) and dry matter content of the leaves were determined using the saturation weighing method, based on fresh weight, saturated fresh weight, and dry weight (Afzal et al., 2017). Peroxidase (POD) activity was determined using the guaiacol method (Sarikurkcu et al., 2016). POD catalyzed the oxidation of guaiacol to form red-brown tetra-methoxyphenol in the presence of H_2_O_2_. The absorbance was measured at 470 nm, and tenzyme activity was calculated based on the rate of increase in absorbance per unit time. Ascorbic acid (AsA) was extracted and quantified using commercial assay kits (Nanjing Jiancheng Bioengineering Institute, Nanjing, China). The Fe³^+^ solution was used to react with reduced AsA to generate Fe²^+^, which subsequently reacted with phenanthroline for absorbance measurement at 536 nm.

### 2.3 Anatomical structure analysis

Anatomical characteristics were analyzed using the paraffin sectioning technique combined with the safranin-fast green staining method. Specifically, 0.5 cm × 0.5 cm tissue blocks were excised from mature leaves, stems, main roots, and lateral roots, immediately fixed in FAA solution (90 mL 50% ethanol, 5 mL glacial acetic acid, and 5 mL formaldehyde) for 24 h. The fixed tissues were then subjected to gradient dehydration, clearing, wax infiltration, and embedding to prepare 8 μm thick paraffin sections. The sections were sequentially dewaxed in xylene, rehydrated in absolute ethanol, rinsed with tap water, stained in safranin solution for 2 h, counterstained in fast green solution for 60s, and finally dehydrated with absolute ethanol. The stained sections were observed using a light microscope (Nikon Eclipse Ci, Japan).

### 2.4 Statistical analysis of data

Excel 2020 software (Microsoft, USA) was used for data analysis. After one-way analysis of variance was used to determine the difference, the Duncan test was used for multiple comparisons.

### 2.5 Hormone and pathway annotation analysis

The endogenous hormones and hormone-related metabolites in sour jujube were quantified using high-performance liquid chromatography-mass spectrometry (HPLC-MS, Cao et al., 2020). The analytes included SA, 1-aminocyclopropanecarboxylic acid, JA, jasmonoyl-isoleine, cis-12-oxophytodienoic acid, ABA, auxin, isopentenyl adenine, isopentenyl adenosine, trans-zeatin, trans-zeatin riboside, cis-zeatin, cis-zeatin riboside, dihydrozeatin, gibberellin A1, gibberellin A3, gibberellin 4, gibberellin 7, castasterone, typhasterol, and brassinolide, totaling 21 compounds.

Detected plant hormones were annotated against the kyoto encyclopedia of genes and genomes (KEGG) database for systematic pathway analysis. The zeatin biosynthesis pathway (ko00908) was specifically reconstructed to illustrate the metabolic network. Key genes involved in zeatin biosynthesis and metabolism were subsequently selected for expression analysis. The heat map was generated using TBtools-II (version 2.310; Chen et al., 2023a).

### 2.6 Transcriptome data acquisition and processing

The raw data of transcriptome analysis were downloaded from NCBI Sequence Read Archive (SRA) database (PRJNA1419927, Li et al., 2026). After quality control and adapter trimming following the filtering criteria described by Song et al. (2017), the clean reads were aligned to the *Ziziphus jujuba* var. *spinosa* reference genome (Li et al., 2024) using STAR (v2.7.10a) with default parameters. Gene expression was quantitatively analyzed through aligned read files by Cufflinks (version 2.2.1). Gene expression levels were calculated by fragments per kilobase per million mapped reads (FPKM, Li and Dewey, 2011).

Differential expression analysis was performed using DESeq2 (Love et al., 2014), with differentially expressed genes (DEGs) identified using thresholds of |Log_2_ fold change (FC)| > 1 and false discovery rate (FDR) < 0.05. Functional annotation of DEGs was performed against the kyoto encyclopedia of genes and genomes (KEGG) database and enrichment analysis was conducted using the clusterProfiler R package (*p* < 0.05).

### 2.7 Gene co-expression network construction and analysis

To identify the key gene modules associated with endogenous hormone content, a co-expression network was constructed using the weighted gene co-expression network analysis (WGCNA) R package (v1.72) based on hormone content and FPKM expression matrix of DEGs (**Supplementary Tables 1**, **S2**). The Pearson correlation coefficient was weighted using a soft threshold power function, an adjacency matrix was constructed and converted into a TOM matrix. The co-expression modules were identified through the dynamic shearing tree method (with minSize set to 30). The correlation coefficients between the feature gene values of each module (ME) and the traits were calculated. Key modules were selected based on |cor| > 0.8 and *P* < 0.05. Candidate genes were screened using kME > 0.98 within the key module. Subsequently, protein-protein interaction network prediction analysis was performed using the STRING database. Gene co-expression networks were visualized with Cytoscape (version 3.10.3).

### 2.8 RNA extraction RT-qPCR analysis

Total RNA was extracted using an RNA Rapid Extraction Kit (Aidlab Biotechnologies Co., Ltd., Beijing, China) according to the manufacturer’s protocol. The integrity and purity of the RNA were assessed by 1% agarose gel electrophoresis. RNA concentration and purity were precisely determined using a NanoDrop 2000 spectrophotometer (Thermo Fisher Scientific, DE, USA). High-quality RNA was then reverse transcribed into first-strand cDNA using a reverse transcription kit (Aidlab Biotechnologies Co., Ltd., Beijing, China).

The qPCR amplification was performed using 2 × SYBR^®^ Green qPCR Mix (Aidlab Biotechnologies Co., Ltd., Beijing, China) on a Roche LightCycler 96 system (Hoffmann-La Roche, Basel, Switzerland). Each reaction was prepared in a total volume of 25 µL. Relative gene expression levels were calculated using the 2^−ΔΔCt^ method (Takahashi et al., 2017). All primer sequences used in this study are listed in **Supplementary Table 3**.

## 3 Results

### 3.1 Phenotypic and physiological responses of sour jujube to ABA and cytokinin treatments under drought stress

**Figure 1:**
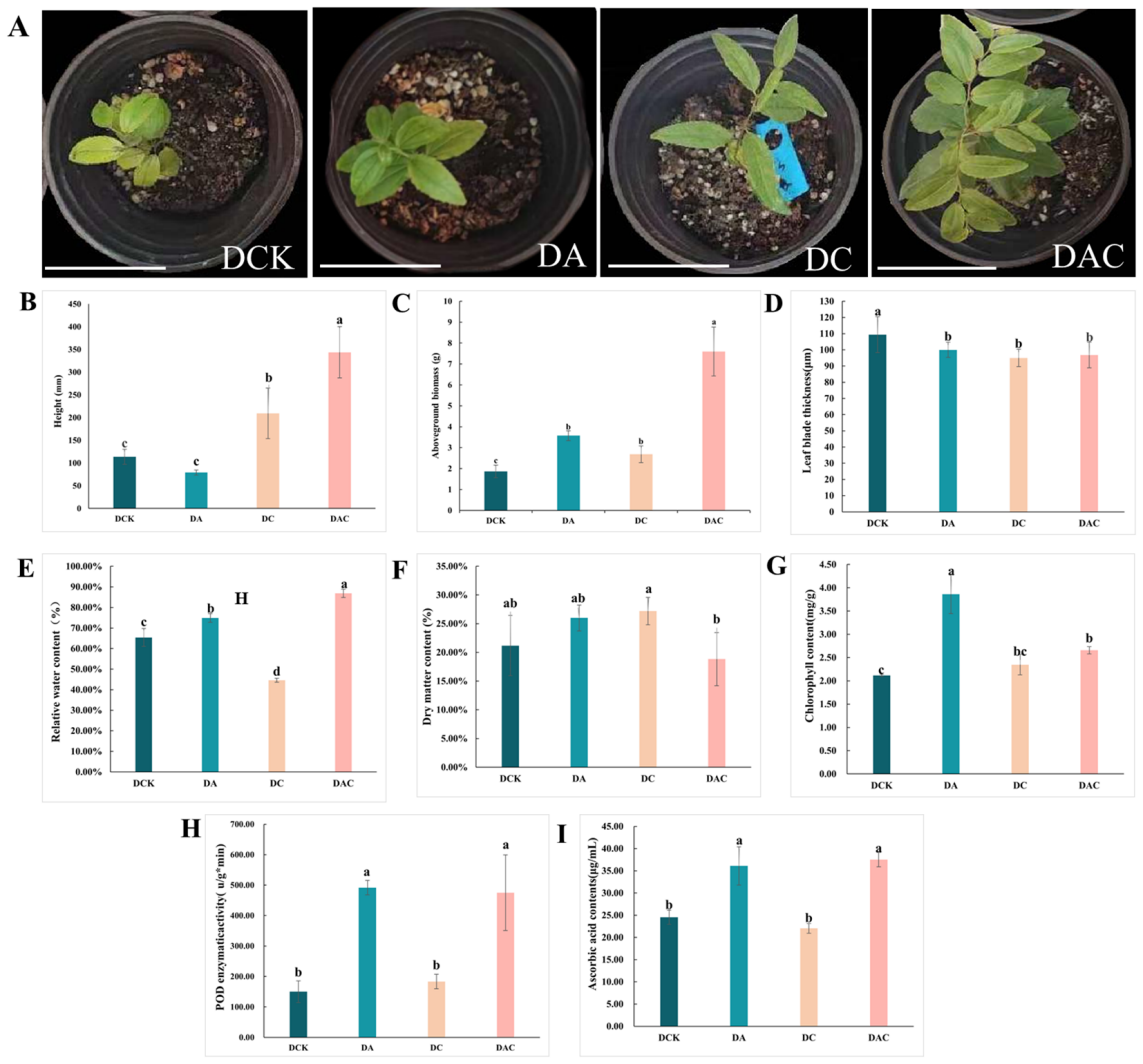
Phenotypic characteristics **(A–D)** and physiological responses **(E–I)** of sour jujube to ABA and cytokinin treatments under drought stress. **(A–D)** Phenotype **(A)** and characteristics **(B–D)** of DCK, DA, DC, DAC: height **(B)**, aboveground biomass **(C)** and leaf blade thickness **(D)**. **(E–I)** RWC **(E)**, dry matter content **(F)**, chlorophyll content **(G)**, POD enzyme activity **(H)** and AsA content **(I)** of DCK, DA, DC, and DAC. Values represent the means ± SD (n=3). Different lowercase letters represent significant differences at the 0.05 level. *Bar* 10.0* cm*.

Comparative analysis revealed that both DA and DAC improved drought resistance relative to DCK, with DAC demonstrating the most pronounced protective effects. Conversely, cytokinin compromised drought resistance, highlighting the negative impact of cytokinin on plant drought resistance when applied in isolation under water deficit conditions. As shown in **Figure 1A**, after drought stress, DCK exhibited leaf yellowing, apical bud wilting, slender stems, and overall weak growth vigor. In contrast, DA displayed a dwarfed phenotype but with thicker stems and stronger growth performance, indicative of ABA-mediated drought adaptation. Notably, DC developed narrower leaves than DCK, along with leaf drooping, yellowing leaf tips, necrotic spots, and complete apical bud death. Their stems and branches were significantly thinner and weaker compared to DCK, suggesting that cytokinin exacerbated drought sensitivity. Additionally, DAC showed enhanced branching and faster vertical growth. The plant height and above-ground biomass of the DAC were 3.02 times and 4.08 times those of the DCK, respectively (**Figures 1B, C**). The results also revealed drought stress led to a decrease in leaf blade thickness after ABA and cytokinin treatments (**Figure 1D**). The DCK exhibited the highest leaf blade thickness, whereas the DA, DAC, and DC showed reductions to 91.4%, 88.4%, and 86.8% of the control levels, respectively, all being significantly lower than DCK (*p* < 0.05).

Further analysis demonstrated that after 14 days of drought treatment, the RWC of leaves in the DAC and DA increased significantly (*p* < 0.05) compared to DCK, reaching 86.83% and 74.86%, respectively, whereas the DC exhibited a notable decline to approximately half of that of DAC (**Figure 1E**). Meanwhile, DC exhibited a significantly increased dry matter content, which was 1.28 times, 1.05 times and 1.44 times that of DCK, DA and DAC respectively (**Figure 1F**). Additionally, as shown in **Figure 1G**, the chlorophyll content followed a consistent trend of DA > DAC > DC > DCK, with DA exhibiting a 35.17% increase compared to DCK. After 14 days of drought stress, POD activity in DA and DAC showed significantly higher compared to that in control (*p* < 0.05), following the order: DA > DAC > DC > DCK (**Figure 1H**). Similarly, DAC and DA increased AsA content by 52.9% and 47.1% respectively, compared to DCK (*p* < 0.05), with the trend: DAC > DA > DCK > DC (**Figure 1I**).

### 3.2 Anatomical traits of leaf vein, root and stem after ABA and cytokinin treatments under drought stress

**Figure 2:**
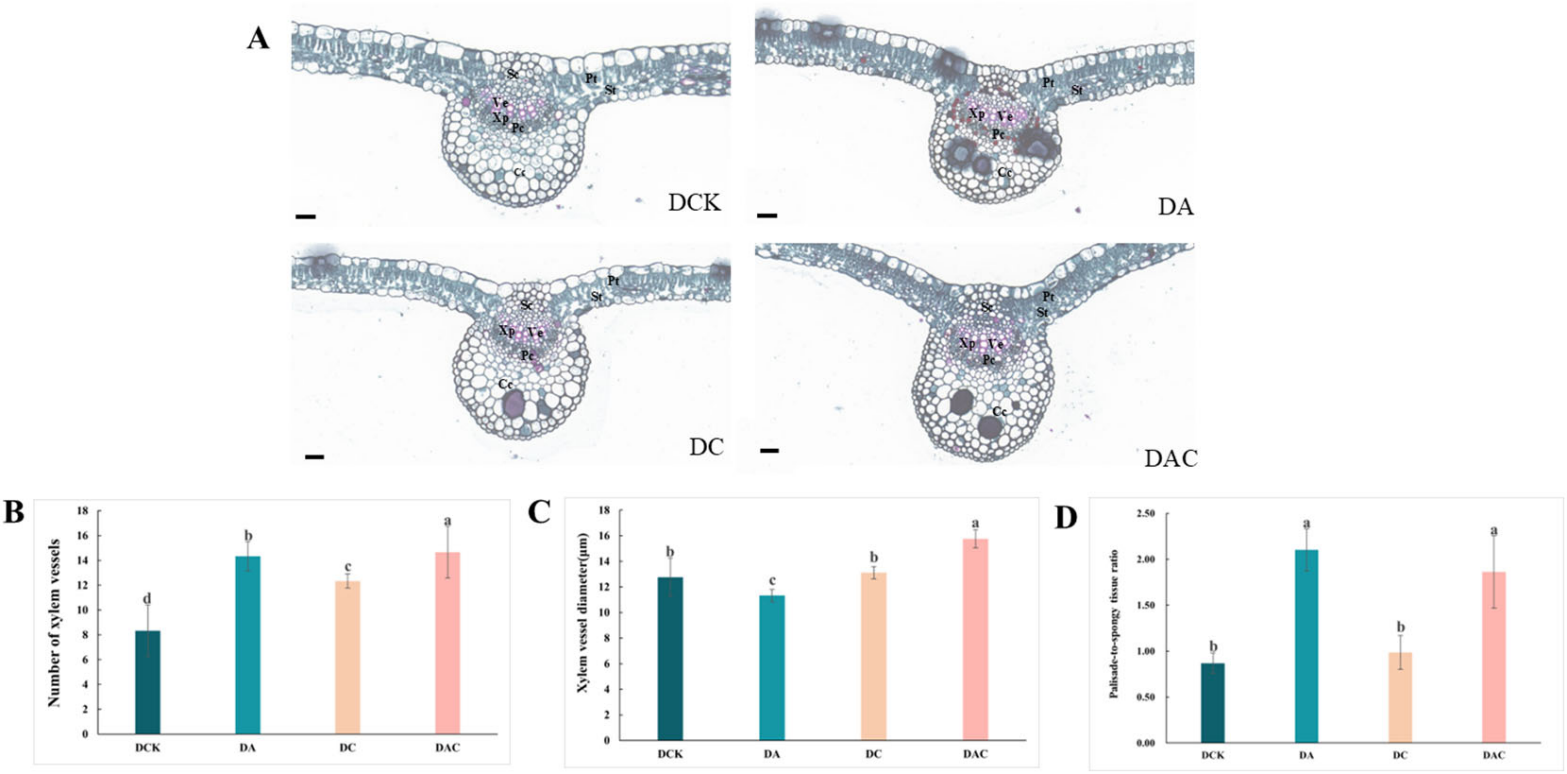
Anatomical analysis of leaves vein of sour jujube treated with ABA and cytokinin under drought stress. **(A)** Transverse section of the leaves vein of DCK, DA, DC, and DAC. Sc, sclerenchyma; Xv, xylem vessel; Xp, xylem parenchyma; Pc, phloem cell; Cc, collenchyma; Pt, spongy tissue; St, palisade tissue. *Bar* 50μm. **(B–D)** Number of xylem vessel **(B)**, xylem vessel diameter **(C)**, palisade-to-spongy tissue ratio **(D)** of DCK, DA, DC, and DAC. Values represent the means ± SD (n=3). Different lowercase letters represent significant differences at the 0.05 level.

Anatomical analysis revealed that ABA and cytokinin significantly altered the vein structure of sour jujube leaves (**Figure 2**). Compared with DCK, the number of xylem vessels of DA increased significantly (*p* < 0.05), and the ratio of palisade tissue thickness to spongy tissue thickness (P/S ratio) increased significantly (*p* < 0.05). However, the diameter of the xylem vessels in DA decreased significantly (*p* < 0.05). In the DC, the number of xylem vessels significantly increased compared with DCK (*p* < 0.05), while vessel diameter remained unchanged. Furthermore, DC did not significantly change the P/S ratio. Notably, the number and diameter of xylem vessels as well as the P/S ratio in DAC were significantly greater than those in DCK (*p* < 0.05).

**Figure 3:**
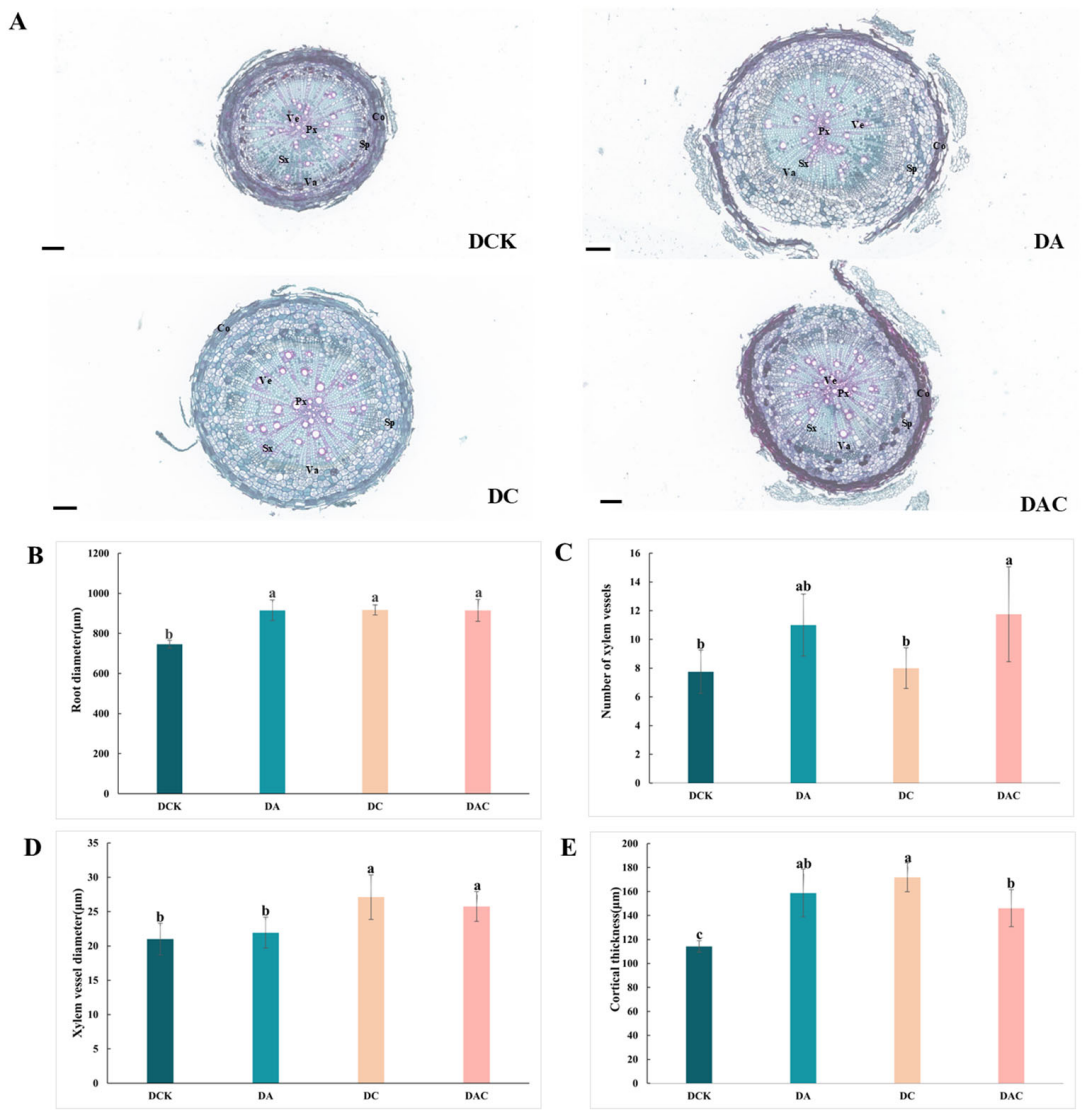
Anatomical analysis of main root of sour jujube treated with ABA and cytokinin under drought stress. **(A)** Transverse section of the root of DCK, DA, DC, and DAC. Px, primary xylem; Sx, secondary xylem; Sp, secondary phloem; Va, vascular cambium; Ve, xylem vessel; Co, cortex. *Bar* 100μm. **(B–E)** Root diameter **(B)**, number of xylem vessels **(C)**, xylem vessel diameter **(D)**, cortical thickness **(E)** of DCK, DA, DC, and DAC. Values represent the means ± SD (n=3). Different lowercase letters represent significant differences at the 0.05 level.

As shown in **Figure 3**, ABA and cytokinin significantly altered the anatomical characteristics of both the main root and lateral roots under drought stress compared to the control. In the main root, DA increased root diameter (*p* < 0.05), and the number of xylem vessels, and significantly increased cortical thickness by 32.26%, while the vessel diameter remained unchanged. On the contrary, DC significantly increased root diameter, xylem vessel diameter, and cortical thickness (*p* < 0.05). DAC also significantly increased root diameter, xylem vessel number, vessel diameter, and cortical thickness (*p* < 0.05).

The anatomical structure of lateral roots was also observed and statistically analyzed (**Supplementary Figure 1**). Compared to DCK, DA showed a significant increase in the number of vessels but a decrease in their diameter. Specifically, the lateral root diameter (*p* < 0.05) was significantly reduced by 85.93%, and the cortical thickness (*p* < 0.05) was significantly reduced by 75.41%. On the contrary, DC significantly increased the diameter of vessels in the lateral root, while the root diameter remained unchanged. DAC slightly increased the number of vessels and increased their diameter. Meanwhile, DAC significantly increased lateral root diameter and secondary xylem thickness (*p* < 0.05), but slightly decreased cortical thickness.

**Figure 4:**
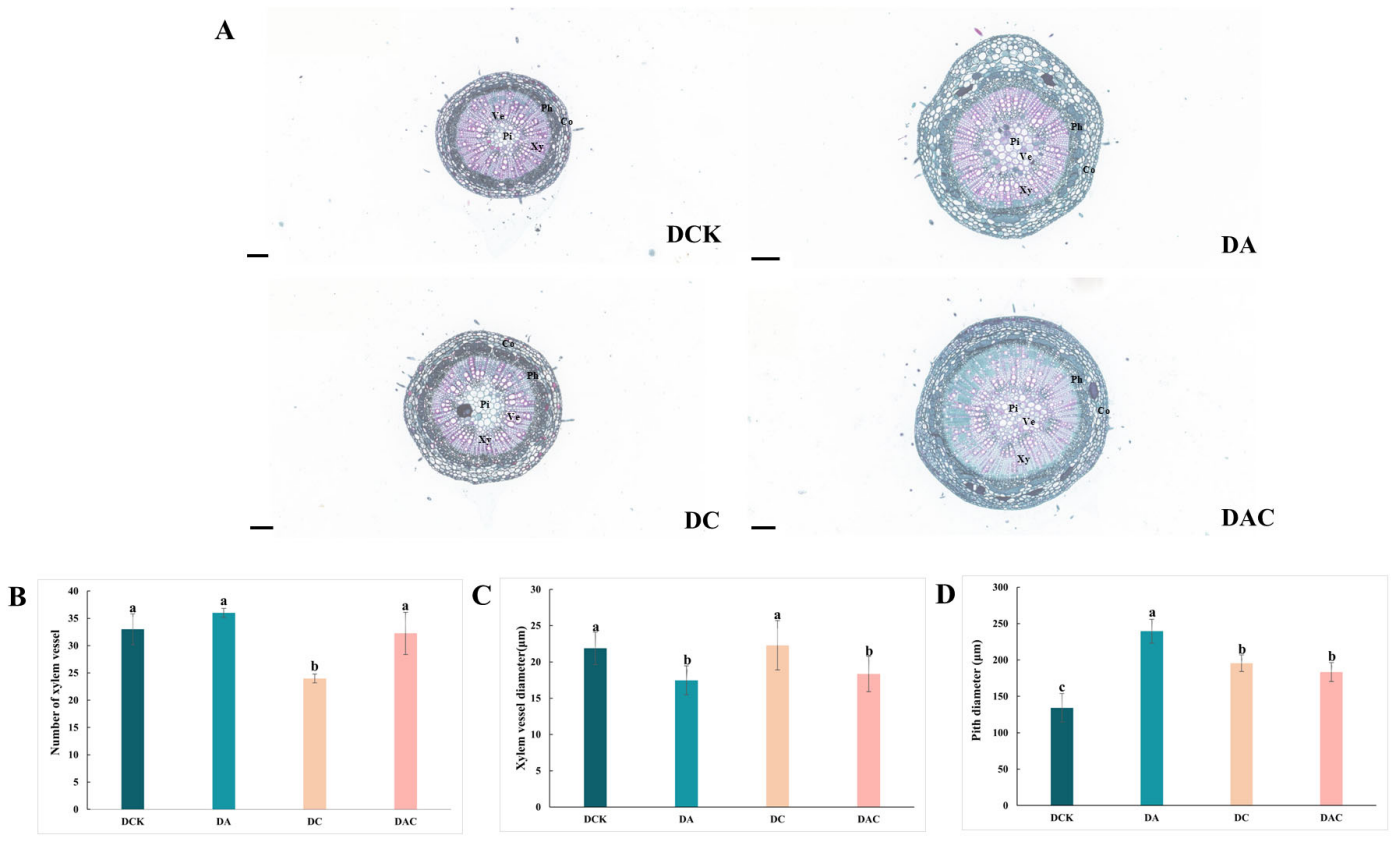
Anatomical analysis of stem of sour jujube treated with ABA and cytokinin under drought stress. **(A)** Transverse section of the stem of DCK, DA, DC, and DAC. Xy, xylem; Pi, pith; Ph, phloem; Ve, xylem vessel; Co, cortex. *Bar* 100μm. **(B–D)** Number of xylem vessels **(B)**, xylem vessel diameter **(C)**, pith diameter **(D)** of DCK, DA, DC, and DAC. Values represent the means ± SD (n=3). Different lowercase letters represent significant differences at the 0.05 level.

There are significant differences in the anatomical structure of stems among all samples (**Figure 4**). The diameter of the xylem vessels in the stem significantly decreased in DA compared to DCK. Notably, the pith diameter in DA exhibited substantial enhancements, rising by 78.38%, compared to DCK. In contrast, DC showed a significant reduction in xylem vessel number and a significant expansion in pith diameter, while maintaining unchanged vessel diameter. For DAC, a significant reduction in vessel diameter and a significant increase in pith diameter were observed.

### 3.3 Endogenous hormone profiles responses of sour jujube to ABA and cytokinin treatments under drought stress

**Figure 5:**
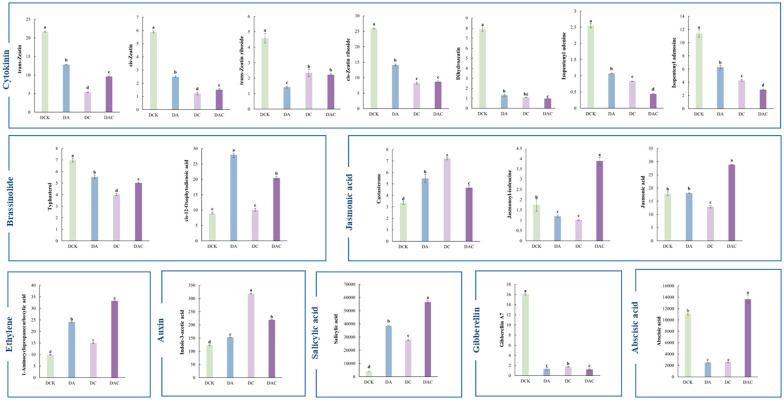
Endogenous hormone profiles of sour jujube in DCK, DA, DC, and DAC. Different lowercase letters represent significant differences at the 0.05 level.

Comprehensive hormone profiling of four samples revealed 17 plant hormones representing eight distinct classes: ABA, ethylene, brassinosteroids, cytokinin, gibberellin, auxin, SA and jasmonic acid (JA). Cluster analysis revealed that ABA and cytokinin treatments significantly altered both the composition and concentration of endogenous hormones in the leaves (**Supplementary Figure 2**). Cytokinin and gibberellins were significantly lower in DA, DC, and DAC than in DCK. Compared to DCK, both DAC and DA significantly elevated SA levels, reaching 14.1-fold and 9.63-fold higher concentrations respectively (**Figure 5**). The DAC accumulated the highest ABA content, followed by the DCK controls at 37.06%, while DA and DC showed no significant differences in ABA accumulation. Notably, DC induced the highest Indol-3-acetic acid concentration, which was significantly greater than DA by 20.41%, DAC by 12.25%, and DCK by 24.2% (*p* < 0.01). Jasmonoyl-isoleucine and JA were significantly up-regulated in DAC, while there was no significant change in DA, and JA was significantly down-regulated in DC.

Additionally, the auxin-to-cytokinin ratio in DCK, DA, DC, and DAC were further analyzed. Compared to DCK, all treatment groups exhibited a pronounced increase in this ratio, where the DC demonstrated the most dramatic elevation with an 8.40-fold increase relative to control levels. Furthermore, both DA and DAC demonstrated substantial increases, reaching 1.40-fold and 5.51-fold of control levels, respectively (**Supplementary Figure 3**). This result indicates that ABA attenuates the cytokinin-induced surge in the auxin-to-cytokinin ratio.

### 3.4 Identification of endogenous hormone response co-expression modules and candidate genes based on WGCNA

**Figure 6:**
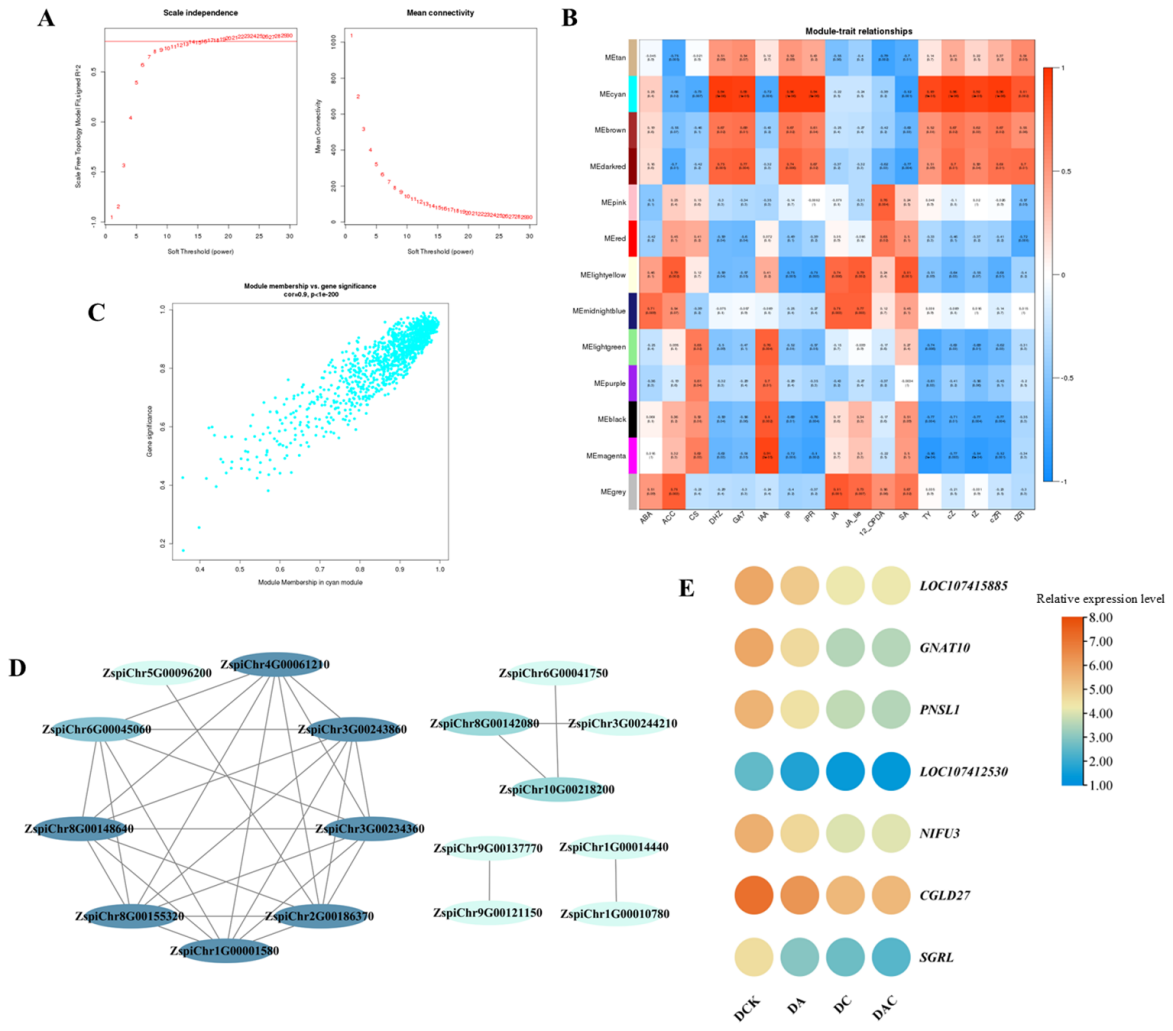
Identification of co-expression modules and candidate genes through WGCNA. **(A)** Network topology analysis of soft threshold. **(B)** Correlation between baits and modules. **(C)** Correlation between module membership and gene significance in the cyan module. **(D)** Protein interaction network in the cycan module. **(E)** Expression patterns of 7 DEGs annotated in the zeatin pathway. The elliptical shape and color represent the number of connections, with darker colors indicating a greater number of connections. The lines connecting the shapes represent the interaction between the proteins encoded by the genes.

WGCNA was performed using endogenous hormone contents as traits (**Supplementary Table 1**) and DEGs as background pools (**Supplementary Table 2**) to screen candidate genes. As shown in **Figure 6A**, when the soft-thresholding power *β* = 16, the network fit index R² reached above 0.85, satisfying the criteria for scale-free network distribution; this power was therefore selected as the optimal parameter for constructing the co-expression network. Gene modules were identified using the dynamic tree-cutting method, and module eigengenes (MEs) were calculated for each module. Modules with similar expression profiles were merged, ultimately yielding 13 co-expression modules, which were distinguished by different colors (**Figure 6B**). Correlation analysis between each module ME and hormone traits revealed that the cycan module exhibited the strongest correlation with the zeatin-related hormone traits (|cor| > 0.8). Further analysis of this module showed a significant positive correlation between module membership (MM) and gene significance (GS, cor = 0.9, *p* < 1e^-200^), indicating that genes with higher intramodular connectivity were more strongly correlated with hormone traits (**Figure 6C**). Based on protein interaction regulation prediction, the regulatory relationship between genes encoded proteins in the cycan module was constructed and visualized (**Figure 6D**). Finally, seven candidate genes related to zeatin signaling pathway were ultimately identified, namely: *LOC107415885* (*Zspichr4G00061210*), *GCN5-related N-acetyltransferase 10* (*GNAT10*, *Zspichr3G00243860)*, *photosynthetic NDH subunit of lumenal location 1* (*PNSL1*, *Zspichr3G00234360)*, *LOC107412530* (*Zspichr2G00186370)*, *NifU-like protein 3* (*NIFU3*, *Zspichr1G00001580)*, *conserved in the green lineage and diatoms 27* (*CGLD27*, *Zspichr8G00155320)*, and *stay-green rice like* (*SGRL*, *Zspichr8G00148640)*. Expression level analysis showed that DCK exhibited the highest transcriptional level in all 7 candidate genes (**Figure 6E**). Among them, *CGLD27* had the highest expression level in DCK, reaching 1.8 times, 3.32 times, and 3.36 times that of DA, DC, and DAC, respectively. In contrast, the expression levels of *GNAT10* in DC and DAC were 81.57% and 81.35% lower than that of DCK, and 58.05% lower in DA. Meanwhile, the expression levels of *LOC107415885* in DA, DC, and DAC were 43.51%, 68.71%, and 68.76% lower than that of DCK, respectively.

### 3.5 Response of the zeatin signaling pathway to ABA and cytokinin treatments under drought stress

**Figure 7:**
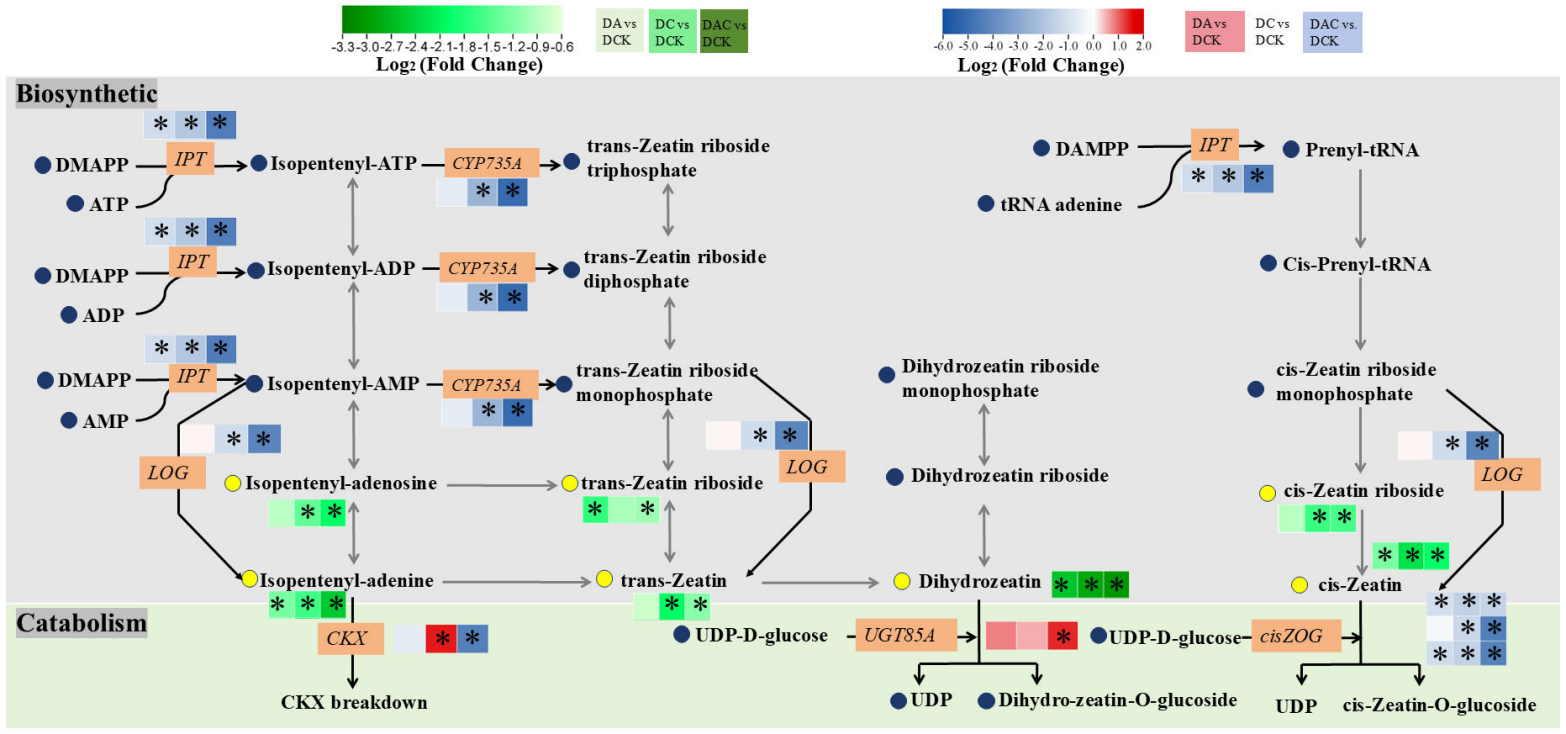
Molecular pathway of zeatin related genes and compounds co-annotation in zeatin biosynthesis (ko00908). The pathway is divided into two sub-pathways: biosynthesis (gray background) and catabolism (green background). Differentially expressed genes and differential accumulated compounds in DA vs DCK, DC vs DCK, and DAC vs DCK are represented by squares, with key gene labeled in orange box. * indicates |log_2_ (fold change)| > 1.

The zeatin biosynthesis and metabolism pathway was further presented based on ko00908 (**Figure 7**). ABA and cytokinin treatment under drought conditions led to a decrease in the contents of zeatin and its intermediates, including trans-zeatin, dihydrozeatin, isopentenyl adenine, cis-zeatin, isopentenyl adenosine, trans-zeatin riboside and cis-zeatin riboside among all the comparison groups. This indicated that ABA and cytokinin treatment inhibited zeatin synthesis and metabolism under drought stress. Therefore, we further examined the expression levels of key genes involved in the biosynthesis and transformation of these compounds: *adenylate isopentenyltransferase* (*IPT, LOC125418705*), *Cytokinin hydroxylase* (*CYP735A*, *ZspiChr4G00063610*), and *cytokinin riboside 5’-monophosphate phosphoribohydrolase* (*LOG*, *LOC107411352*), as well as genes involved in zeatin metabolism regulation: *UDP-glycosyltransferase 85A* (*UGT85A*, *ZspiChr12G00250420*), *Cytokinin dehydrogenase* (*CKX*, *ZspiChr6G00037160*), *zeatin O-xylosyltransferase 1* (*ZOG1*, *ZspiChr3G00233690*), *zeatin O-glucosyltransferase 2* (*ZOG2*, *ZspiChr3G00233710*), and *Zeatin O-glucosyltransferase 3* (*ZOG3*, *ZspiChr12G00248440*) in DCK, DA, DC, and DAC. Further RT-qPCR analysis demonstrated that the expression levels of genes related to trans-zeatin metabolism were upregulated, while the expression levels of genes involved in trans-zeatin synthesis and cis-zeatin synthesis and metabolism were significantly downregulated. Moreover, the fold changes of the seven genes were the highest in DAC vs. DCK compared to DA vs. DCK and DC vs. DCK, suggesting that ABA and cytokinin co-application might induce unique response characteristics during the zeatin biosynthesis. The expression levels of the synthesis and activation-related genes *IPT* and *LOG* were most downregulated in DAC, with 21.0-fold and 17.8-fold downregulation compared to DCK, respectively. This trend was similar to the 4.8-fold and 2.9-fold downregulation of isopentenyl adenine and isopentenyl adenosine contents in DAC vs. DCK. Under drought conditions, compared to DCK, the glycosylation-related gene *UGT85A* was significantly upregulated by 1.7-fold in DA and 2.6-fold in DAC. The cytokinin oxidase/dehydrogenase gene *CKX* was significantly upregulated by 1.7-fold in DC but showed no significant change in DA and was significantly downregulated by 19.5-fold in DAC. However, the isopentenyl adenine in DC and DAC were increased degradation. Meanwhile, the reduced expression of the *LOG* in DC and DAC suggested decreased synthesis of isopentenyl adenine. The three cisZOG-encoding genes *ZOG1*, *ZOG2*, and *ZOG3* exhibited the same response mechanism but showed different levels of variation. Among them, *ZOG2* showed no significant difference in DA vs. DCK, which might lead to the smaller downregulation fold of cis-zeatin in DA vs. DCK compared to DC vs. DCK and DAC vs. DCK.

## 4 Discussion

Phytohormones play pivotal roles in mediating plant drought stress responses (Seleiman et al., 2021). Our study systematically evaluated the effects of exogenous ABA and cytokinin on drought resistance through integrated phenotypic, physiological, hormonal and molecular analyses. The results demonstrated that ABA treatment significantly enhanced drought resistance, whereas cytokinin exhibited inhibitory effects. Notably, the combined treatment of ABA and cytokinin markedly improved drought resistance (**Figure 1**), suggesting a complex hormonal crosstalk under stress conditions. Further investigation revealed that the zeatin signaling pathway serves as a key regulatory node during ABA and cytokinin co-mediated drought adaptation, with critical regulatory genes identified within this pathway.

### 4.1 Phenotypic and physiological mechanisms of ABA induced drought resistance in sour jujube seedlings

ABA regulates plant stress responses through multiple mechanisms, including osmotic adjustment, enzymatic activation, and drought-responsive gene expression (Papacek et al., 2017). Under drought stress, sour jujube seedlings exhibited severe growth inhibition, characterized by leaf chlorosis, apical bud wilting, and stem thinning typical symptoms of drought-induced growth arrest and cellular damage (Rashid et al., 2024). In contrast, sour jujube after ABA treatment displayed a compact yet robust phenotype with vigorous growth, suggesting that ABA orchestrates morphological and physiological remodeling to enhance drought adaptation (Guo et al., 2025). ABA treatment significantly improved leaf RWC, likely through stomatal regulation to maintain water balance under stress. Anatomical evidence demonstrates that ABA reshapes xylem architecture by increasing vessel density in both leaf veins and roots while preserving root vessel diameter but reducing that of leaf veins, a modification that may optimize hydraulic efficiency (Ramachandran et al., 2020; Zhu et al., 2024). Concurrently, the elevated palisade-to-spongy tissue ratio in sour jujube leaves after ABA treatment likely enhanced water-use efficiency and photosynthetic capacity (Zhai et al., 2022), a trait previously documented in drought-adapted apple (Griffith and Einhorn, 2023) and tomato (Scartazza et al., 2017). Notably, ABA also stimulated chlorophyll biosynthesis and AsA accumulation, aligning with findings in other species (Sharma et al., 2024). Furthermore, SA functions as a pivotal defense hormone, the accumulation of SA in sour jujube seedlings after ABA treatment suggests that ABA may prime stress resistance by activating SA-mediated signaling pathways (Wani et al., 2017). Collectively, these results demonstrate that ABA enhances drought resistance in sour jujube through integrated phenotypic adjustments and antioxidant system reinforcement.

### 4.2 Phenotypic and physiological mechanisms by which cytokinin reduce drought resistance in sour jujube seedlings

Cytokinin is a central phytohormone that orchestrates crop morphology, physiology, and yield potential by integrating stem-cell proliferation, inflorescence development, plant architecture, nitrogen-use efficiency, and environmental stress resilience (Zhao et al., 2024). Our findings demonstrate that cytokinin application significantly compromised drought resistance in sour jujube, aligning with prior reports that cytokinin may downregulate immune-related genes via the CRE1 pathway, thereby attenuating plant stress resilience (Laffont et al., 2015). In contrast to ABA treatment, cytokinin application exacerbated drought-induced symptoms—including leaf narrowing, necrosis, and apical bud mortality—suggesting cytokinin accelerate senescence under water deficit, consistent with established cytokinin-mediated senescence pathways (Markovich et al., 2017). Notably, cytokinin induced organ-specific structural modifications: xylem vessel diameter was markedly enlarged in roots, whereas it remained unaltered in leaf veins and stems. This differential regulation likely reflects tissue-specific cytokinin signaling mechanisms, potentially disrupting water uptake and allocation strategies (Sinclair and Friml, 2019; Xia et al., 2021). Seedlings exhibited excessive stem elongation with reduced stem diameter compared to control. This result may reflect hormonal redistribution and metabolic reprogramming, contributing to xylem structural remodeling (Kakani et al., 2009). Previous studies have suggested that cytokinin-induced stress responses can activate auxin biosynthesis via ROS signaling (Kolachevskaya et al., 2017) and promote IAA3 multimerization (Roy et al., 2025). It was explained that the increased auxin to cytokinin ratio after cytokinin treatment (**Supplementary Figure 3**) may have activated the increased auxin synthesis (**Figure 5**). Collectively, cytokinin application during drought requires caution, as morphological impairments (e.g., weakened stems and imbalanced hormone ratios) may ultimately exacerbate water stress susceptibility.

### 4.3 Combined effects of ABA and cytokinin in enhancing drought resistance in sour jujube seedlings

The combined application of ABA and cytokinin promoted vigorous branching and improved growth performance in sour jujube, suggesting that cytokinin and ABA likely interact through crosstalk to regulate downstream signaling networks (Chen et al., 2023b), thereby enhancing vegetative growth under drought stress. Cytokinin and ABA co-treated seedlings exhibited significantly increased leaf RWC, indicating improved water retention capacity. This enhancement can be attributed to optimized stomatal regulation as well as structural modifications in leaf and root tissues (Liu et al., 2024). Anatomical analysis revealed that co-treatment with cytokinin and ABA induced the increase and enlargement of xylem vessels in leaf veins and taproot, increased palisade-to-spongy mesophyll ratio, and thickened the taproot cortex, collectively improving hydraulic conductivity and water-use efficiency (Li et al., 2022b). These structural adaptations likely constitute the primary basis for superior drought resistance. Furthermore, cytokinin and ABA co-treated seedlings showed elevated POD activity and AsA content, demonstrating effective activation of the antioxidant defense system to scavenge drought-induced ROS and mitigate oxidative damage (Li et al., 2022a). Crucially, our findings highlight that the reprogramming of endogenous hormone homeostasis represents a key factor in enhanced drought resistance. The hormonal profile of seedlings after cytokinin and ABA treatment characterized by high SA, JA levels and increased ABA proportion-indicates a reconstructed signaling network that triggers multifaceted defense responses. Elevated ABA may directly activate drought-responsive gene expression (Liu et al., 2022), while SA and JA accumulation enhances systemic stress resistance (Li et al., 2025; Wang et al., 2022). In conclusion, our results demonstrate that the combined treatment of ABA and cytokinin enhances drought resistance in sour jujube through integrated morphological, physiological, and hormonal adjustments. This multi-faceted mechanism offers new insights for developing improved drought resistance strategies in woody plants.

### 4.4 Regulation of ABA and cytokinin on zeatin signaling under drought stress

Zeatin, as a natural cytokinin, has significant functions in regulating plant growth, development, and adaptation to adverse conditions (Zhao et al., 2025). In this study, 7 candidate genes related to zeatin signaling were selected. These genes are involved in hormone signal transduction, metabolic regulation and stress response processes, suggesting that they may play a role in the zeatin regulatory and drought adaptation (Ishikawa et al., 2020; Ivanauskaite et al., 2023; Sakuraba et al., 2014). The expression analysis revealed that these candidate genes in the control group were higher than those in the other treatment groups, indicating that ABA and cytokinin inhibited their expression under drought stress. *CGLD27* balanced the intermediate and final products of tetra-pyrrole to avoid photo-oxidative damage in *Arabidopsis thaliana* (Zhang et al., 2021); while in this study, the expression level of *CGLD27* in the control treatment was significantly higher than that in ABA and ABA-cytokinin treatments, indicating that exogenous hormones may affect the endogenous hormone balance by altering the transcriptional activity. Additionally, *GNAT10* in *Arabidopsis thaliana* and *Malus baccata* may be participate in DNA damage repair and signaling and stress response (Fina and Casati, 2015; Rodriguez et al., 2013). The downregulation fold change of *GNAT10* and *LOC107415885* in cytokinin and ABA-cytokinin treatments was greater than that in the ABA treatment, suggesting that cytokinin may no longer merely serves as a growth-promoting signal, but instead acts as a synergistic amplification factor for the ABA inhibitory signal.

The nearly complete suppression of zeatin synthesis and metabolism pathways under drought stress—evidenced by drastic reductions in metabolic flux—suggests a strategic growth arrest in sour jujube seedlings to conserve energy for drought resistance. This inhibition was particularly pronounced following ABA or combined ABA-cytokinin treatments, which triggered a shutdown of zeatin translocation mechanisms. Conversely, cytokinin may represent an alternative mechanism to manage zeatin excess rather than pathway suppression. Notably, ABA and cytokinin co-application already downregulated zeatin synthesis in seedlings, with no evidence of compensatory pathway activation. This was corroborated by parallel decreases in pathway-related gene expression and detectable hormone levels, indicating a coordinated suppression of zeatin metabolism. Under drought, ABA-cytokinin co-application specifically upregulated *UGT85A*, suggesting synergistic promotion of zeatin glycosylation to modulate trans-zeatin homeostasis and enhance drought acclimation (Chen et al., 2021; Jin et al., 2013; Zhao et al., 2025). Remarkably, the downregulation of *CKX* under combined ABA-cytokinin treatment may confer drought resilience by reducing transpiration, boosting photosynthesis, and enhancing antioxidant capacity (Hu et al., 2025; Rashid, et al.,2024), potentially explaining the observed increase in stem diameter and drought resistance. Compared to cytokinin treatment, ABA application led to higher accumulation of cis-zeatin via *ZOG1*, *ZOG2* and *ZOG3*, which may delay ABA-induced leaf senescence (Chen et al., 2020; Martin et al., 2001; Wang et al.,2023) and further improve drought resistance. These findings raise the intriguing possibility that ABA promotes stem thickening while cytokinin induces thinning (Hura et al., 2017), a hypothesis warranting mechanistic exploration in future studies to elucidate the hormonal regulation of stem architecture under drought.

## 5 Conclusions

In this study, phenotypic, anatomical, physiological, and hormone content characterizations were used to analyze the differences in sour jujube seedlings treated with ABA and cytokinin under drought conditions. Our findings demonstrate that ABA treatment produced dwarfing but robust sour jujube seedlings by promoting xylem proliferation in both leaf veins and roots, and elevating SA content, thereby improving drought resistance. Cytokinin markedly compromised drought resistance, inducing plant frailty, enlarging root xylem-vessel diameter, and elevating auxin levels. Co-application of ABA and cytokinin produced thicker plants with more branches, increased both the number and diameter of xylem vessels in leaf veins and taproots, and markedly elevated SA, ABA and JA levels while reinforcing the antioxidant system, resulting in significantly enhanced drought resistance. In addition, the zeatin-related compounds were selected to screen candidate genes through WGCNA, *CGLD27*, *GNAT10* and *LOC107415885* were found be regulated by ABA and cytokinin. Further analysis of zeatin signaling pathway, we also found that both ABA and cytokinin reduced zeatin synthesis, but increased trans-zeatin metabolism and decreased cis-zeatin metabolism. The study provides a theoretical basis for the study of plant drought resistance mediated by plant hormones, and provides a new perspective for the stress resistance breeding of jujube rootstocks. Future research should combine field experiments to validate core traits, and use methods such as genetic transformation and gene editing to deeply reveal the functions and molecular mechanisms of the candidate genes in hormone crosstalk regulation of drought resistance.

## Data Availability

The datasets presented in this study can be found in online repositories. The names of the repository/repositories and accession number(s) can be found in the article/**Supplementary Material**.
