# Supplementary material for: Exogenous ABA combined with cytokinin enhances drought resistance in Ziziphus jujuba Mill. var. spinosa by regulating xylem development and zeatin signaling pathway
Source: Front Plant Sci. 2026 Jul 30;17:1901882. doi: 10.3389/fpls.2026.1901882 (PMC13470638; doi:10.3389/fpls.2026.1901882)
Supplement: Supplementary file 1 [file Supplementaryfile4.docx]

| **Gene name** | **Forward** | **Reverse** |
| --- | --- | --- |
| *ZjActin* | AGCCTTCCTGCCAACGAGT | TTGCTTCTCACCCTTGATGC |
| *LOC107416560* | GCCCCTGCATTTATGGGAGA | CGTAGCAGCTGTCGAACTCT |
| *LOC125418705* | GAGGGTGGGACCTACAGAGA | ACTCCACCTCTGCAAACTGT |
| *LOC107431889* | CGTGACTTTGGCATTTTCAGGT | TATCAATTATGGCGGCCTCG |
| *LOC107420171* | TCTGTTTTGAATGAGGACTCG | TGGAATCGGTTTTGCGAACA |
| *LOC107411352* | ACCTGGTGGTTATGGGACAC | TGGAGTAGGGGCAGAGACAA |
| *LOC107413439* | AGCCATTCGAACCAGCTCTT | TGTTGGGTCTTGGATGAAAGG |
| *LOC107431606* | GTGGAGCTTCTCGCATCGAA | GGTTGTACAGCTTCTAGGCCC |
| *LOC107413455* | TCCAGCTTCGAATTCTACTGA | GGTGGGTTGGAAAACGTAGG |
|  |  |  |

Supplementary Table S3 Primers used in the study
