# Supplementary material for: Exogenous ABA combined with cytokinin enhances drought resistance in Ziziphus jujuba Mill. var. spinosa by regulating xylem development and zeatin signaling pathway
Source: Front Plant Sci. 2026 Jul 30;17:1901882. doi: 10.3389/fpls.2026.1901882 (PMC13470638; doi:10.3389/fpls.2026.1901882)
Supplement: Supplementary file 2 [file Supplementaryfile1.doc]

**Figure S1** Anatomy analysis of lateral root of sour jujube treated with ABA and cytokinin under drought stress. (A-E) The number of xylem vessels A), Root diameter(B), cortical thickness(C), xylem vessel diameter (D), secondary xylem(E) of DCK, DA, DC, and DAC. Different lowercase letters represent significant differences.

**Figure S****2** Cluster analysis of endogenous hormone content in DA vs DCK (A), DC vs DCK (B) and DAC vs DCK (C).

**Figure S3** The auxin / cytokinin ratio of DCK, DA, DC and DAC.
